# Increased Abundance of Cytotoxic T Cells in Synovial Fluid of a Dog With Erosive Immune‐Mediated Polyarthritis

**DOI:** 10.1155/crve/9048633

**Published:** 2026-04-29

**Authors:** Kohei Murakami, Kosuke Kobayashi, Kenji Kutara, Shin-ichi Nakamura, Michihito Tagawa, Takamasa Itoi

**Affiliations:** ^1^ Faculty of Veterinary Medicine, Okayama University of Science, Imabari, Ehime, Japan, ous.ac.jp

## Abstract

Most dogs with nonerosive immune‐mediated polyarthritis (IMPA) have neutrophilic inflammation in the synovial fluid. On the other hand, some dogs with erosive IMPA have increased mononuclear cells as the most abundant cell subset in the synovial fluid. A 9‐year‐old, neutered female miniature dachshund presented with lameness and lethargy. Physical examination revealed subluxation of the carpal and tarsal joints. Synovial fluid tests revealed an increased number of leukocytes, mainly mononuclear cells, and radiographic examination led to a diagnosis of erosive IMPA. Compared with nonerosive IMPA, flow cytometric analysis of the synovial fluid in this case showed a significant expansion of T cells—predominantly CD8^+^ cytotoxic T cells—while monocyte and B cell counts remained comparable. Treatment with mycophenolate mofetil reduced leukocytes in the synovial fluid and improved the dog′s activity, although bone erosion slowly progressed. Based on these findings, we propose that T cells, especially cytotoxic T cells, in synovial fluid contribute to inflammation in canine erosive IMPA and may be a therapeutic target.

## 1. Introduction

Immune‐mediated polyarthritis (IMPA) is characterized by an increased white blood cell (WBC) count in synovial fluid from multiple joints. IMPA is classified into two broad categories, erosive and nonerosive, based on whether destructive changes in bone are evident in radiographs [1]. In erosive IMPA, radiographic changes occur in joints of the extremities, and destruction of supportive ligaments may lead to joint instability and luxation [2, 3]. In contrast to novel therapeutic advances in treating human rheumatoid arthritis, the pathogenesis of erosive IMPA in dogs is poorly understood, and therapeutic development has not progressed.

Most dogs with IMPA, whether erosive or nonerosive, have neutrophilic inflammation in the synovial fluid [4, 5], and lymphocytes are not the main cellular subset. In erosive IMPA, however, lymphocytes occasionally increase in the synovial fluid and may become the most abundant cell subset [6]. The number of lymphocytes in the synovial fluid of dogs with erosive IMPA is significantly higher than in dogs with nonerosive IMPA [7], indicating that lymphocytes may be involved in erosive changes in canine IMPA, but it remains unclear which types of lymphocytes contribute to the pathogenesis. Here, we propose that T cells, especially cytotoxic T cells, in synovial fluid contribute to canine erosive IMPA.

## 2. Case Presentation

A 9‐year‐old female miniature dachshund presented for evaluation of lameness in all four limbs and reduced mobility. Eleven months before presentation, the dog had been seen by the referring veterinarian due to her reluctance to move. At that time, intervertebral disc disease was suspected, and conservative, short‐term treatment was provided.

**Figure 1:**
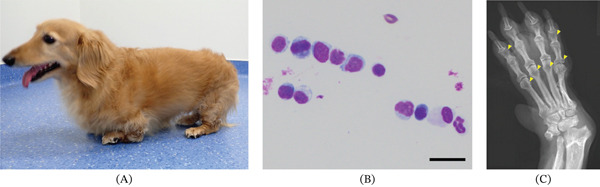
Increased abundance of mononuclear cells in the synovial fluid of a dog with erosive immune‐mediated polyarthritis. (A) Carpal and tarsal hyperflexion in the present case. (B) Increased cellularity, mainly mononuclear cells, in the synovial fluid. Cells were stained with Diff Quick. Scale bar, 20 *μ*m. (C) Dorsopalmar radiographs of the left manus. Subluxation of the carpal joint and decreased opacity of epiphyses adjacent to affected joints (arrowhead) were observed.

On presentation, the dog walked on the whole soles of the feet (plantigrade) (Figure 1A). Physical examination revealed hyperextension and hyperflexion of carpal and tarsal joints. The dog had a rectal temperature of 39.1°C, and the normal size of superficial lymph nodes was noted. A complete blood count (CBC) revealed mild polycythemia (Hgb 19.2 g/dL, reference range 12.0–18.0 g/dL) and normal WBC (6600/*μ*L, reference range 6000–17,000/*μ*L) and platelet (350 × 10^3^/*μ*L, reference range 200–500 × 10^3^/*μ*L) counts. A serum chemistry panel—including glucose, blood urea nitrogen, creatinine, aspartate aminotransferase, alanine aminotransferase, total protein, albumin, electrolytes (sodium, potassium, and chloride), calcium, inorganic phosphorus, and creatine phosphokinase—revealed no abnormalities. The C‐reactive protein level was also within the reference range (0.05 mg/dL, reference range < 1.0 mg/dL).

Synovial fluid was obtained from the carpal (right and left) and tarsal (left) joints. The synovial fluid was slightly increased in quantity (0.2–0.5 mL per joint) and appeared watery and turbid. The viscosity was markedly decreased, as confirmed by a string test result of < 1 cm. Cytological examination of stained smears revealed high cellularity in all samples, and the total nucleated cell counts ranged from 5200 to 8160 cells/*μ*L (evaluated by hemocytometer). Neutrophils, lymphocytes, and large mononuclear cells composed 13%–16%, 55%–60%, and 24%–32% of nucleated cells, respectively (Figure 1B). Cytological evaluation clarified that neither microorganisms nor degenerative neutrophilic changes were seen. Bacterial cultures were not performed due to the small amount of synovial fluid. Radiographs of carpal and tarsal joints revealed periarticular osteoporosis, characterized by increased radiolucent areas at epiphyses of phalanges (Figure 1C). Systemic evaluation, including CBC, serum chemistry, and thoracic/abdominal CT imaging, revealed no evidence of underlying triggers such as occult infection or neoplasia. These findings effectively excluded secondary IMPA. Because radiographic erosive changes, as observed in the present case, are characteristically limited to either Greyhound polyarthritis or erosive IMPA [3], a diagnosis of erosive IMPA was established.

To clarify the pathogenesis, we assessed the dog′s leukocyte composition in peripheral blood and synovial fluid by flow cytometry. The peripheral blood and synovial fluid from each of the left carpal and left tarsal joints were obtained following a diagnosis of erosive IMPA. Nine synovial fluid samples from six dogs with nonerosive IMPA (three females and three males) were also enrolled as a comparison group with a mean age of 5.3 (range 3.5–8.9) years. Breeds examined included toy poodles (*n* = 4), a miniature dachshund (*n* = 1), and mixed (*n* = 1). The inclusion criteria for nonerosive IMPA were the presence of neutrophilic inflammation in synovial fluid samples from two or more joints, with a complete absence of radiographic evidence of bone erosion in the specific joints where inflammation was identified. In these nonerosive IMPA cases, potential underlying triggers were excluded based on a CBC, a comprehensive biochemical profile, and imaging of the thorax (orthogonal radiographs or CT) and abdomen (ultrasound or CT). A comprehensive PCR panel for tick‐borne diseases was also performed to rule out infections, including *Anaplasma* spp., *Ehrlichia* spp., *Leishmania* spp., and *Rickettsia* spp., if the dog had a recent history of travel outside Japan or a recent detection of ticks. The study protocol was approved by the ethical review committee of the Veterinary Medical Teaching Hospital of Okayama University of Science (approval number, 2022‐010).

For preparation of samples for flow cytometry analysis, EDTA‐treated peripheral blood samples were treated with ammonium–chloride–potassium (ACK) lysis buffer to remove red blood cells. Synovial fluid samples were washed with phosphate‐buffered saline (PBS) and subjected to cell staining. Cell surface staining for flow cytometry analysis was performed using fluorescence‐activated cell sorting (FACS) staining buffer (1× HBSS with 2% FBS). Before cell staining, GhostDye Violet 510 (Cytek Biosciences, Fremont, California, United States) was added to cell suspensions to exclude cellular debris and dead cells. Prepared cell samples were incubated for 10 min with an Fc receptor–binding inhibitor polyclonal antibody (14‐9162‐42; eBioscience, San Diego, California, United States) to prevent nonspecific staining.

**Figure 2:**
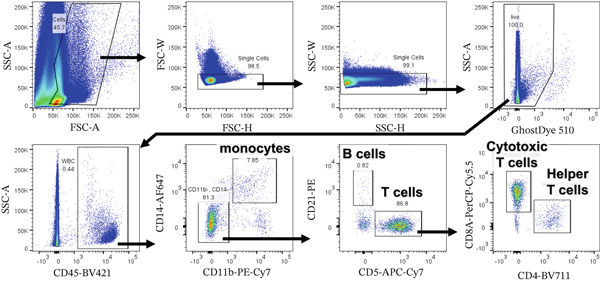
Flow cytometry gating strategy for peripheral blood and synovial fluid analysis. Representative flow cytometry plots of synovial fluid from a dog with erosive IMPA.

Peripheral blood and synovial fluid were stained for 20 min on ice with fluorescent dye–conjugated monoclonal antibodies specific for the following markers: CD45 (clone YKIX716.13, 48‐5450‐42; eBioscience), CD11b (clone M1/70, 101216; BioLegend, San Diego, California, United States), CD14 (clone TÜK4, MCA1568A647; Bio‐Rad Laboratories, Hercules, California, United States), CD21 (clone CA2.1D6, MCA1781PE; Bio‐Rad), CD5 (clone YKIX322.3, 47‐5050‐42; eBioscience), CD4 (clone YKIX302.9, 67‐5040‐42; eBioscience), and CD8a (clone YCATE55.9, 46‐5080‐42; eBioscience). Stained cells were analyzed using a BD LSR Fortessa X‐20 cell analyzer (BD Biosciences, San Jose, California, United States). All flow cytometry data were analyzed using FlowJo software v10.9 (BD Biosciences). The flow cytometry gating strategy for peripheral blood and synovial fluid analysis is shown in Figure 2.

In synovial fluid from dogs with nonerosive IMPA, median B cell and T cell counts were 4/*μ*L and 82/*μ*L, respectively. In the present case, B cell counts in the synovial fluid of the left carpus and tarsus were 0.4/*μ*L and 35/*μ*L, and T cell counts were 4895/*μ*L and 3672/*μ*L, respectively (Table 1). This indicates remarkable T cell–mediated inflammation within the erosive joints. Furthermore, the increased T cells in the left carpus and tarsus were predominantly CD8^+^ cytotoxic T cells (44.7% and 58.6% of CD45^+^ leukocytes) rather than CD4^+^ helper T cells (8.8% and 8.9% of CD45^+^ leukocytes; Table 1). Compared with dogs with nonerosive IMPA, numbers and percentages of cytotoxic T cells and helper T cells in synovial fluid were higher, although B cell and monocyte counts were not. Cytotoxic T cells were abundant in the synovial fluid from affected joints compared to peripheral blood, whereas helper T cells, CD21^+^ B cells, and CD11b^+^CD14^+^ monocytes in the synovial fluid were similar or less abundant than in peripheral blood.

**Table 1:** Leukocyte composition in dogs with immune‐mediated polyarthritis (IMPA) numbers of CD21^+^ B cells, CD5^+^ T cells, CD4^+^ helper T cells, CD8^+^ cytotoxic T cells, and CD11b^+^CD14^+^ monocytes and their percentages among CD45^+^ cells. Nine synovial fluid (SF) samples from 6 other dogs with nonerosive IMPA were also examined and are shown with median and 95% confidence intervals (CIs).

	Dogs with nonerosive IMPA	The present case with erosive IMPA		
	SF	Carpus SF	Tarsus SF	Whole blood
	Cells/*μ*L (%)	Cells/*μ*L (%)	Cells/*μ*L (%)	Cells/*μ*L (%)
	Median [95% CI]			
B cells	4 [0.7–11.6] (0.1 [0.1–0.4])	0.4 (0.01)	35 (0.7)	334 (6.0)
T cells	82 [−5.3–505.5] (3.0 [−0.01–11.4])	4895 (60.0)	3672 (70.6)	728 (11.0)
Helper T cells	30 [7–137] (1.3 [0.3–3.1])	715 (8.8)	462 (8.9)	265 (4.7)
Cytotoxic T cells	30 [−26–345] (0.8 [−0.9–7.7])	3651 (44.7)	3049 (58.6)	109 (1.9)
Monocytes	479 [81–1621] (14.2 [8.0–28.5])	998 (12.1)	408 (7.9)	742 (11.0)

Since T cell–mediated autoimmunity was suspected, the dog was treated with cyclosporine (Atopica; Novartis, 5 mg/kg, bid) for 4 weeks, but the number of leukocytes in synovial fluid from the left carpus showed only a minimal decrease (3840 nucleated cells/*μ*L). Then, cyclosporine was replaced with mycophenolate mofetil (MMF) (Cellcept; Chugai, 10 mg/kg, bid). Six weeks later, leukocytes had almost disappeared from the synovial fluid from the left carpus (125 nucleated cells/*μ*L), and the dog had become more active. The dog was monitored monthly for clinical signs and underwent blood and synovial fluid tests every 3 months. Although radiographs obtained 7 months later showed slight progression of erosive changes in the medial aspect of the distal radius and the radial carpal bone, neither inflammatory cell infiltration in the synovial fluid nor systemic clinical signs have recurred for more than 1 year.

## 3. Discussion

In dogs with erosive IMPA, there are numerous CD5^+^ T cells diffusely and perivascularly distributed in the inflamed synovial tissue [8]. In the present case, while initial treatment with cyclosporine was ineffective, switching to MMF, an inosine monophosphate dehydrogenase (IMPDH) inhibitor, successfully controlled the joint inflammation. Since MMF inhibits the *de novo* pathway of purine synthesis in both T and B lymphocytes, its clinical efficacy suggests that B cells, in addition to T cells, may play a significant role in the pathogenesis of erosive IMPA. However, it should be noted that therapeutic drug monitoring for cyclosporine was not performed; therefore, the possibility that the treatment failure was due to subtherapeutic blood levels cannot be ruled out. Despite the clinical improvement and the marked reduction of lymphocytes in the synovial fluid under MMF therapy, bone erosion continued to progress slightly. This indicates that cells other than lymphocytes, such as synoviocytes, may also contribute to the bone erosive process in this case.

It has been reported that a large number of CD4^+^ cells are present in the synovial tissue and synovial fluid of dogs with IMPA [8, 9]. However, since canine neutrophils also express CD4 [10], we need to carefully interpret the results where multicolor staining has not been performed. Considering clinically normal canine synovial fluid contains 13.6% CD4^+^ cells and 16.5% CD8^+^ cells [11], the erosive joints of the present case had an obvious accumulation of CD8^+^ cytotoxic T cells. Cytotoxic T cells recognize peptide fragments that are bound to major histocompatibility complex class I molecules and kill cells infected with intracellular pathogens. In addition, cytotoxic T cells are involved in autoimmune diseases, especially organ‐specific autoimmune diseases such as Hashimoto′s thyroiditis and multiple sclerosis [12]. In human rheumatoid arthritis, CD8^+^ T cells are known to increase in the synovial fluid while remaining at normal levels in peripheral blood [13]. These synovial CD8^+^ T cells abundantly express the anti‐inflammatory cytokine IL‐10, and their frequency is inversely correlated with disease activity, suggesting a regulatory or suppressive role in human rheumatoid arthritis [13]. In contrast, in the present case, the proportion of lymphocytes in the synovial fluid decreased as the patient′s clinical condition improved. This suggests that the CD8^+^ T cells in this dog may have functioned as proinflammatory effector cells rather than the regulatory phenotype described in human rheumatoid arthritis. Further research is needed to determine whether this increase in cytotoxic T cells and their potential effector function is a characteristic feature of canine erosive IMPA.

## Author Contributions

Kohei Murakami: conceptualization, validation, investigation, writing (original draft), visualization, project administration, and statistical analysis. Kosuke Kobayashi: investigation and writing (review and editing). Kenji Kutara: investigation and writing (review and editing). Shin‐ichi Nakamura: investigation. Michihito Tagawa: investigation and writing (review and editing). Takamasa Itoi: conceptualization, writing (review and editing), and supervision. All intellectual content, data interpretation, and conclusions were developed by the authors, who take full responsibility for the final version of the manuscript.

## Funding

This study was partially funded by the Japan Society for the Promotion of Science, 10.13039/501100001691, 24K09276.

## Ethics Statement

Informed consent to use collected clinical information for research and publication purposes was obtained.

## Conflicts of Interest

The authors declare no conflicts of interest.

## Data Availability

The data that support the findings of this study are available from the corresponding author upon reasonable request.
